# Quality of generic medicines in South Africa: Perceptions versus Reality – A qualitative study

**DOI:** 10.1186/1472-6963-12-297

**Published:** 2012-09-03

**Authors:** Aarti Patel, Robin Gauld, Pauline Norris, Thomas Rades

**Affiliations:** 1 Southern African Development Community (SADC), PO Box 95, Gaborone, Botswana; 2 Hera: right to health & development, Reet, Belgium; 3 Department of Preventive and Social Medicine, University of Otago Medical School, PO Box 913, Dunedin 9054, New Zealand; 4 New Zealand's National School of Pharmacy, University of Otago, PO Box 56, Dunedin 9054, New Zealand

## Abstract

**Background:**

Generic Medicines are an important policy option allowing for access to affordable, essential medicines. Quality of generic medicines must be guaranteed through the activities of national medicines regulatory authorities. Existing negative perceptions surrounding the quality of generic medicines must be addressed to ensure that people use them with confidence. Campaigns to increase the uptake of generic medicines by consumers and providers of healthcare need to be informed by local norms and practices. This study sought to compare South African consumers’ and healthcare providers’ perceptions of quality of generics to the actual quality of selected products.

**Methods:**

The study was conducted at the local level in three cities of South Africa: Johannesburg, Durban and Cape Town. Purposive sampling was used to recruit consumer participants (n = 73) and random sampling used to recruit healthcare providers from public and private sectors (n = 15). Data were obtained through twelve focus group discussions with consumers and semi-structured interviews (n = 15) with healthcare providers in order to gain familiarity with perceptions of quality. One hundred and thirty five products comprising paracetamol tablets (n = 47), amoxicillin capsules (n = 45) and hydrochlorothiazide tablets (n = 43) were sourced from public and private sector healthcare providers. These products were subjected to *in vitro* dissolution, uniformity of weight and identity (Fourier Transformed Infrared Spectroscopy) tests using prescribed methods from the British (2005) and United States Pharmacopeias (2006).

**Results:**

Respondents described drug quality in relation to the effect on symptoms. Procurement and use behavior of healthcare providers was influenced by prior experience, manufacturers’ names and consumers’ ability to pay. All formulations passed the *in vitro* tests for quality.

**Conclusions:**

This study showed clear differences between perceptions of quality and actual quality of medicines suggesting deficiencies in public engagement by government regarding the implementation of generic medicines policy. Implementation of generic medicines policy requires the involvement of consumers and healthcare providers to specifically address their information gaps and needs.

## Background

Generic medicines policies are used by governments to improve access to affordable medicines. Successful uptake of this policy is dependent on society having confidence in the efficacy, safety and quality of generic medicines [1,2]. Together with medicines promotion, we know that commercial features of medicines like price and brand names affect peoples’ perceptions of efficacy and safety [3]. Additionally, there is a prevailing notion that generic medicines are inferior in quality [2]. These perceptions are further reinforced by recent studies that found increasing numbers of poor quality medicines in the developing world [4,5]. Whilst the issue of poor quality medicines has to be urgently addressed, negative perceptions about generic medicines must be managed through proper engagement of all stakeholders in those settings where the regulatory environment delivers quality-assured medicines [2,6,7].

A medicine is considered to be poor quality when it does not meet established standards in terms of identity, purity, bioavailability of the active pharmaceutical ingredient and appropriate packaging and labeling of the finished product [8]. This description frames quality within a technical paradigm. Studies assessing quality tend to focus on measuring drug content; limited research is undertaken on how the concept of quality is understood by the general public and healthcare providers [9]. Consumers have been shown to rely on the advice of their healthcare providers [10,11], with providers relying on experience, pharmaceutical marketing, prior use and clinical outcomes when making decisions about which medicines to use [3,10]. Increasingly, we recognize that the use of medicines is influenced by shared experiences and knowledge informed by local contexts [12,13].

South Africa introduced the National Medicines Policy in 1996. Whilst generic medicines have always been part of the system, the focus has mainly been in the public sector. From mid 2000, when medicine prices become a critical issue, generic medicines have been recommended for wider use, including the private sector. The objectives of this study were to explore consumers’ and providers’ perceptions of quality of medicines, assess the quality of selected essential medicines and ultimately compare the perceptions of quality to the actual quality of the products tested. The findings, showing a disconnect between perceptions of quality to the actual quality, indicate that implementation of the generic medicines policy in South Africa requires a multi-pronged approach that is developed through the participatory mechanisms that pay attention to the social, economic and cultural contexts of the intended beneficiaries, i.e. the users and the providers.

## Methods

The study was conducted in three South African cities, Durban, Johannesburg and Cape Town, from 2006 to 2008. Qualitative methods, consisting of focus group discussions with seventy-three consumers, and interviews with fifteen healthcare providers, were used to gain familiarity with users’ and providers’ views on quality of medicines as well as generic medicines. Participants were recruited through purposive sampling. Twelve focus groups - four in Johannesburg, five in Durban and three in Cape Town - were conducted with consumers from different ethnicities, ages, gender and socio-economic status. Recruitment of consumer participants followed a two stage process. Initially eleven key informants were identified from existing networks developed by the first author through her prior work in South Africa. These key informants then recruited the remaining consumer participants through snowball sampling. Table 1 describes the characteristics of the consumer participants.

**Table 1:** Description of consumer participants

***City***	***Focus group***	***No. of people***	***Gender***	***Age***	***Employment (formal/informal)***
Johannesburg	1	6	Female	20 – 60	informal
	2	6	Male	20 – 48	informal
	3	6	Male (3)	28 – 63	formal
			Female (3)		
	4	7	Male (5)	25 – 56	Formal
			Female (2)		
Durban	5	6	Male	28 – 48	Informal
	6	6	Male (4)	27 – 54	Formal
			Female (2)		
	7	6	Female	37 – 64	Formal
	8	8	Male	68 – 87	Retired
	9	5	Male	16 – 19	Students
Cape Town	10	6	Female	25 – 53	Informal
	11	6	Male (3)	27 – 38	Formal
			Female (3)		
	12	5	Female	65 – 73	Retired

Informal Employment: domestic worker, driver, cleaner, security.

Formal Employment: police, teacher, lawyer, admin officer.

Fifteen semi-structured interviews were conducted with healthcare providers from both the private and public health sectors in South Africa. Private sector providers were randomly selected within a particular geographic location. This involved drawing up a list of medical practices and community pharmacies in the suburb where the first author stayed during the data collection period. The list was informed by local residents together with scoping missions during field visits. Letters introducing the study were drafted and delivered by local contacts to a total of twelve practices that consisted of six medical practices and six pharmacies. The idea was to approach a minimum of two medical practices and two pharmacies, per suburb, to interview the doctors, nurses and pharmacists. Letters introducing the project were sent out and permission sought to interview the respective providers. Three pharmacy and three medical practices granted us permission for interviews. Two of the medical practices were dispensing doctor practices. The three pharmacies consisted of one chain store pharmacy and two independent pharmacies. Through this process eight private practitioners were recruited: three general practitioners, two nurses and three pharmacists. For the public sector, provincial staff, known to the first author from her previous work in South Africa, were approached and asked to participate. Two pharmacists, three doctors and two nurses were included from the public sector. Table 2 describes the characteristics of these providers.

**Table 2:** Description of healthcare providers

***City***	***Provider***	***Public***	***Private***
Johannesburg	Doctor	1	1
	Nurse	1	1
	Pharmacist	1	2
Durban	Doctor	1	-
	Nurse	1	-
	Pharmacist	1	1
Cape Town	Doctor	1	2
	Nurse	-	-
	Pharmacist	-	1

The aim of the focus group discussions with consumers and interviews with providers was to gather insights into peoples’ understanding of quality and use of generic medicines rather than reaching consensus on quality of generic medicines. Therefore, due to time and cost limitations, the above approaches to recruitment of participants were adopted.

The following issues for both the consumers and healthcare providers were explored during the group discussions and interviews:

– Describe your understanding of the quality of a medicine?

– Where do you get your medicines from? Why do you use this source?

– Which product would you use to treat a headache (shown two versions of paracetamol: *Panado*^*R*^ (original) and *Pacimol*^*R*^ (generic)?

– Which product would you use to treat an infection (shown two versions of amoxicillin: *Amoxil*^*R*^ (original) and *Moxypen*^*R*^ (generic)?

All focus group discussions and interviews were tape-recorded, transcribed by an assistant and coded with the aid of NVivo, version seven. Initial coding was predominantly open and performed by the first author. During coding three distinct themes were emerging: quality was understood in relation to effects on clinical symptoms, acceptability and use of generic medicines was based on product names, prices paid or source of supplier. After coding according to these themes, the data were then reviewed individually by the others. The analysis was shared with the participants (e-mailed back to the healthcare providers and summarized with focus group participants at the conclusion of group discussions). Their feedback was incorporated into the final analysis.

*In vitro* analysis was performed on 135 samples of selected products that consisted of paracetamol tablets, hydrochlorothiazide tablets and amoxicillin capsules. These three medicines are on the national essential medicines list and are used to treat pain (paracetamol), hypertension (hydrochlorothiazide) and infection (amoxicillin) at primary care level in South Africa [14]. They are available in both the private and public sectors as tablets, capsules and suspensions. The patents on all these medicines have expired and many branded generic versions are available in the country. The originator brand of paracetamol in South Africa is *Panado*^*R*^*.* For amoxicillin, this is *Amoxil*^*R*^ . Both amoxicillin and hydrochlorothiazide, classified as prescription medicines, require a medical prescription when being purchased in South Africa. Paracetamol can be bought over the counter without a prescription.

For this study we obtained our sample of products from sources commonly used by the consumers. Consumers identified three main sources for medicines: the public sector (clinic or hospital), dispensing doctors, and community pharmacies. For the public sector medicines from eleven clinics and hospitals in Durban, KwaZulu-Natal were procured after permission was granted by the provincial Department of Health. Dispensing doctors in Durban and Johannesburg were approached, told about the study, and asked to provide medicines. Medicines were supplied by seven doctors, four in Durban and three in Johannesburg. Medicines were purchased for cash, using prescriptions written by a South African medical colleague, from thirty community pharmacies: eleven in Durban, ten in Johannesburg and nine in Cape Town. Here the data collector started at a central point in the respective city and went to pharmacies within a 20 kilometer radius until approximately ten sites were visited in a single day to purchase the medicines. Medicines were supplied in patient-ready packs with the brand decided by the supplier.

Twenty-nine generic medicines were sourced from the public sector. The remaining 106 samples were sourced from the private sector (dispensing doctors and community pharmacists). Of these, six were samples of Panado^R^ and four were samples of Amoxil^R^, representing innovator brands. Therefore, out of the total of 135 samples, ten were innovator brands supplied solely by the private sector and the remaining 125 samples were branded and unbranded generics from both the public and private sectors. Table 3 provides a description of the medicines assessed in this study.

**Table 3:** Description of medicines tested

***Drug***	***Brands/Name on label***	***Public clinic or hospital (no. of samples)***	***Community Pharmacy (no. of samples)***	***Dispensing Doctor (no. of samples)***
Paracetamol 500 mg tablets	Paracetamol	1	11	1
	Panado	-	5	1
	Painblok	6	2	-
	Pacimol	3	5	-
	Painamol	-	4	1
	Prolief	-	1	3
	Paramed	-	1	-
	Gencetamol	-	1	1
Hydrochlorothiazide 25 mg tablets	Adco-retic*	-	-	3
	Hydrochlorothiazde	2	-	1
	Ridaq	9	22	-
	Hexazide	-	6	-
	*combination product			
Amoxicillin 250 mg capsule	Amoxicillin	-	2	-
	Amoxcil	-	-	4
	Allmox	-	-	1
	Betamox	-	7	1
	Ipcamox	2	1	
	Maxcil	-	1	-
	Moxypen	2	13	-
	Moxymax	1	-	-
	Ranmoxy	-	1	-
	Xeracil	-	1	-
	Zoxil	-	2	-
Amoxicillin 500 mg capsule	Amoxicillin	-	1	-
	Moxypen	3	1	-
	Maxcil	-	-	1

Several tests can be performed on a medicine to determine its quality; tests for identity, content, disintegration, dissolution, stability, sterility, impurity, bioavailability and bioequivalence as per the monographs in the relevant pharmacopeias. This study focused on assessing the identity, dissolution and uniformity of weight. Identity testing is crucial because this verifies the active pharmaceutical ingredient. Fourier transformed infrared spectroscopy (FTIR) was used to assess the identity of the products according to the monographs of the 2005 British Pharmacopeia (BP) for amoxicillin rehydrate 250 mg and 500 mg capsules, hydrochlorothiazide 25 mg tablets, and paracetamol 500 mg tablets. Amoxicillin (Sigma-Aldrich Chemie GmbH; Steinheim, Germany, Lot.112 K0481), Hydrochlorothiazide (Sigma-Aldrich Chemie GmbH; Steinheim, Germany; Lot 115 K1112) and Paracetamol (4-Acetamidophenol) 9Sigma-Aldrich Chemie GmbH; Steinheim, Germany; Lot 11 K0253) were the drug standards used in the tests.

Dissolution tests were performed to determine the rate and amount of active drug going into solution in a specified medium, following the specifications of the drug monographs of the BP, 2005. Apparatus II (paddle apparatus) was used for the paracetamol 500 mg tablets, hydrochlorothiazide 25 mg tablets and amoxicillin 500 mg capsules; Apparatus I (basket apparatus) was used for the amoxicillin 250 mg capsules. The amount of drug going into solution at the respective times was measured using ultraviolet spectrophotometer and compared to the specifications of the BP, 2005.

Uniformity of weight of the formulation was also assessed as a proxy measure of quality control during the manufacture, packaging and distribution of the product. The tablets and capsules were analyzed according to the percentage deviation as recommended in the BP, 2005.

Ethical approval for this study was granted by the Universities of Otago, New Zealand, and KwaZulu-Natal, South Africa.

## Results

### Understanding quality

For consumers, the main descriptor of quality was the effect of the medicine on their symptoms. Terms used to describe quality included “drug works” and “strong medicine”. The following statements capture the general theme of associating the quality of a medicine with the effect it produces on symptoms:

*“Quality is like a standard: low, medium, high. Low standard medicines equal something that doesn’t work. Is about the way it works, side-effects of it, effects of it”* (Johannesburg, group 1).

*“The only way I can define quality is if I drink it and see it works”* (Cape Town, elderly).

Similar to the consumers, healthcare providers also described quality of medicines in relation to alleviation of symptoms felt by their patients or side-effects produced.

*“Quality essentially has to do with how effective and how safe your product is for a patient”* (Pharmacist - private).

*“Quality would first mean efficacy. Secondly it must have minimal side-effects. Lastly it must be consumable”* (Doctor – private).

### Use of generic medicines

The majority of our consumers did not seem to be knowledgeable about generic medicines. In terms of self-treatment with over the counter medicines for minor ailments, such as a headache, consumers tended to identify with Panado^R^, the innovator brand, rather than Pacimol^R^, a branded generic. Panado^R^ was a familiar brand, it was well advertised, and people had used this before where they had experienced relief of their symptoms. On the other hand, few respondents had seen Pacimol^R^, the branded generic.

*“Panado is popular, used this before. It is the first time I am seeing this* (Pacimol)” (Johannesburg, group 2).

*“It (Panado) has been recommended by a lot of people, see it advertised”* (Durban, group 1).

In terms of acceptability of generics regarding prescription medicines, consumers generally tended to rely on the advice of their prescriber, rather than the dispenser.

*“I think if a doctor is prescribing it and he says the generic will be equally potent then I will use it. If I took a script for something and the pharmacist suggested a generic and the doctor had not told me I could use it, then I would be hesitant”* (Cape Town, group 1),

At the same time, generics were acceptable to those consumers who had chronic illnesses and where cost played a deciding role in medicines use.

*“We actually go looking for generics because it is cheaper and we are huge consumers of medicines”* (Durban, group 2).

The healthcare providers all felt that the quality of medicines in South Africa was good. They reported that their selection of generic products was based on prior experiences with a particular product in terms of its efficacy. The message was a clear one of choosing medicines that were tried and tested either by them or their colleagues:

*“You’re seeing 15 – 20 patients a day and you’re using the drugs for that certain period over the 5 or 10 or 15 years and it works. So essentially it’s the effectiveness of the drug that I base my stuff on”* (Doctor – private).

The dispensing doctors mentioned two key factors influencing their use of generic medicines: cost to the patient; and name of the company supplying the medicines:

*“It also depends on the patient profile where you’ve got an elite patient that will ask for the Panado* (a brand name drug) *whereas a B or C category patient I would use a generic, like amoxicillin”* (Doctor - private).

*“I am happy to use generics if it’s from a reputable company. There are some companies in SA where you don’t actually know what or how safe that drug is and if you are going to get reproducible results every time you use it”* (Doctor - private).

Apart from the reputation of the company, which appears to have been assessed through experience with their products, some providers had negative perceptions about products supplied by companies from India and China:

*“If you say to me something’s coming from China, you think about China as being inferior quality in general, with everything else and you wonder from a medical point of view do you actually trust this type of medical, and pharmaceutical industry and developments?*” (Nurse - private).

*“I went to the launch of a product by Dr Reddys. It was incredibly well done. But the problem is I think with the name. If it comes from the East you tend to trust it less. If it comes from Germany, Switzerland you tend to trust it more. So basically where I think they need to either dispel those myths through education”* (Pharmacist - private).

Similarly, consumers felt that generic medicines were inferior and referred to these as being “*fong kong*”, a term used to describe fake goods in Johannesburg.

Managerial interventions were also instrumental in promoting the use of generic medicines. Public sector prescribers were restricted to using what was on their institution’s medicines list. Prescriptions were also written using non-proprietary names.

*“I always use generics for prescribing. Our department doesn't allow prescribing using trade names. We use what is available from PMSC* (Medicines’ depot). *The tender changes intermittently so we stick to generics to avoid confusion”* (Doctor - public)

Below is the description by one of the private sector respondents regarding how financial incentives were used to ensure pharmacists within their chain of pharmacies dispensed specific generics:

*“What’s happened now at head office is they have preferred products. Probably because of kickbacks, we don’t have that much information. But on the system you’ll get Augmentin*^*R*^*, if you choose Augmentin*^*R*^*for an example, and then they will say the first preferred product, the second preferred product, the third preferred product etc… What they’ve now started doing is, if you dispense ‘x’ amount of their preferred products, you get a kickback – the pharmacist, each pharmacist, if you get within a certain percentage. It amounts to about R1000* (~$137) *a month”* (Pharmacist - private).

### Quality concerns

Concerns about the actual quality of medicines were explicitly raised by the public sector pharmacists interviewed. These two pharmacists worked from the provincial medicines’ depots where medicines were received from the suppliers and then distributed to health facilities. They dealt directly with product quality. They reported concerns that medicines intended for use at primary care facilities were not monitored as effectively as more expensive drugs. One said that complaints regarding quality concerns were not adequately dealt with either by the provincial procurement agency or the Medicines Control Council. This created frustration since staff at health care facilities were asked to report drug quality problems but when there was limited feedback, or worse when the supplier was awarded the tender again, it was difficult to remain motivated:

*“Despite numerous reports these companies still get the tender. The sad part is the hospital pharmacists, nursing personnel and patients question the tenders being awarded to such suppliers! My response is “It’s not in my hands”* (Pharmacist - public).

Consumers also acknowledged their limitations in actually assessing quality of medicines and tended to use the price they paid as a key measure of quality. Cheaper medicines were considered to be inferior and people viewed these with a degree of suspicion.

*“Can I ask something from a layman’s point of view? Why are generics cheaper than originals? They make generics for the people who cannot afford the expensive ones or they are just making quick money?”* (Johannesburg, group 2).

*“No, I am thinking in my mind, why this one (*Pacimol*) is R2 (0.30 cents) but is the same as Panado? If I do not have money, I will buy it but…(shakes her head)*” (Johannesburg, group 1)

### *In vitro* analysis of medicines

Out of the 135 preparations tested all samples passed the test for identity. Twelve samples did not pass the phase one of the dissolution testing. There were insufficient tablets within these samples to proceed to phase two dissolution testing. Therefore, results for dissolution testing were positive and conclusive for 123 samples. One hundred and thirty two samples passed the uniformity of weight tests. Physical inspection of these samples showed breakages that probably occurred during the transport of the samples to the laboratory from South Africa. The results were positive for all samples tested. Therefore, there do not appear to be any differences between products sourced from the private or public providers or between innovator brands, branded generics or unbranded generics.

## Discussion

The findings of this study must be considered within its limitations. These were the small sample size of both the products tested and the number of interview and focus group participants. Purposive sampling of the consumer participants and reliance on local knowledge for the drafting of the list of medical and pharmacy practices, has probably biased the study respondents towards the urban and peri-urban settings of South Africa. Time and financial constraints forced us to be pragmatic and adopt our sampling approaches. The study findings, however, highlight issues that were common to all participants that may well be indicative of the perceptions of the wider population. In this sense, further research informed by the findings of this study with a larger group of respondents would be useful. The findings also suggest limitations in the implementation of the generic medicines policy by both government and generic manufacturers as far as providers and consumers are concerned, including lack of information for consumers and an apparent lack of sensitivity in dealing with quality complaints from healthcare providers. Additional detail on South African consumer perceptions of quality of medicines is presented by the authors in another publication [11].

Our findings regarding the *in vitro* tests provide evidence that the products assessed from South Africa meet the quality standards as established in the British and United States Pharmacopeia. The two innovator medicines assessed in this study, Panado^R^ and Amoxil^R^, were both supplied by private sector providers. All samples from the state sector were branded or unbranded generic products. We found no major differences in the quality of products provided by the state sector when compared to those supplied by the private sector, or between innovator and generic brands. These *in vitro* findings are much more positive when compared to other, similar studies undertaken in Africa [15,16].

The findings regarding peoples’ perceptions demonstrate a marked consistency between consumers and healthcare providers in their description of quality of medicines: their concerns are about the safety and efficacy of the medicine on relief of symptoms. From this perspective all respondents generally agreed that the quality of medicines in South Africa was good. We found a clear difference between what consumers perceive as being inferior medicines and the actual quality of medicines. Similarly, whilst most healthcare providers were more positive about the quality of generic medicines, there were certain reservations about quality expressed by pharmacists working in the state sector. These reservations were informed in a large part by the lack of feedback provided to these pharmacists after product quality complaints were made.

Additional reservations against generic medicines by healthcare providers appeared to be driven by internally held beliefs about products that were manufactured in eastern countries such as India and China. These respondents felt that, in general, products from these countries were inferior and transferred this perception to medicines produced there as well. The link to the East was also evident when consumers from Johannesburg referred to perceived fake medicines as “fong kong”. However, Schumaker and Bond’s research in Zambia suggests that such attitudes about products from the East are beginning to change [17]. They found increasing acceptance, by healthcare providers, of generic antiretroviral medicines from India because these were shown to improve health outcomes and were readily available [17].

Suspicion was directed at generic medicines as demonstrated by the majority of participants selecting Panado^R^, the original brand, over Pacimol^R^, a generic. Panado^R^ was familiar and its effects were known to them; Pacimol^R^ was the unknown. The need to engage the public through the provision of trustworthy information about generic medicines should allow for greater acceptance [2].

Understanding the disconnect between providers’ and consumers’ perceptions of quality in comparison to the actual quality probably lies in the subject domains within which medicines and their actions are interpreted. Medicines are chemical entities that have been developed into dosage forms allowing people to consume them and gain the benefits of the active drug [18]. When people consume medicines they experience the effects of them. The dominant paradigm governing this experience is the pharmacological one [19]. Therefore, when measuring the quality of medicines the focus is on the biochemical properties of the drug. The pharmacological paradigm fails to explain other aspects that form part of the medicine use experience, especially differences in the description of effects by the patient compared to those of the clinician or even the placebo effect [20,21]. More specifically, the pharmacological approach fails to explain why some consumers and healthcare providers felt that the cheaper, generic medicines were inferior because these took too long to work. Understanding the experiences and perspectives of those who consume medicines requires policymakers, implementing agencies and researchers to address some of the inadequacies of the purely biomedical perspective and to explore more fully the socio-cultural framework within which medicines use occurs [1,13].

South Africa has legislation and regulations to support the use of generic medicines. This study shows that the gap appears to lie in how these strategies have been communicated to consumers and healthcare providers. The South African government, through the National Medicines Policy and the Essential Medicines Programmed has recommended that tertiary institutions introduce concepts like generic medicines, essential drugs and standard treatment guidelines into undergraduate medical, nursing and pharmacy curricula [22]. These concepts must be embraced by all: students, academics, professional councils and associations to ensure there is a common understanding and for common messages to be shared with the public regarding quality and safety of generic medicines. Also, recent reports about the challenges faced by South Africa’s Medicines Regulatory Authority further fuel negative perceptions around quality of generic medicines and needs to be addressed [23]. Finally, the potential for savings from the use of generic medicines is widely recognized by policymakers so communicating this to the South African public remains an important issue [24].

## Conclusion

The implication from our study is that there needs to be a wider acceptance by governments that medicines do have a “social origin”, and consumers’ and providers’ perceptions must explicitly be addressed if the ultimate goal of improving access to quality assured, affordable medicines is to be realized. In Africa, where access to medicines remains critical, it is timely to start understanding and addressing the problems within the social paradigm [1,10,11] as well as building trust in the agencies responsible for regulation quality and safety of medicines.

## Competing interests

The authors have no competing interests to declare.

## Authors’ contributions

The study was designed by all four authors. Field work, laboratory testing and initial analysis was undertaken by AP. RG and PN reviewed the qualitative findings. TR reviewed the *in-vitro* results. The paper was initially drafted by AP, with review and additions by the others. All authors read and approved the final manuscript.

## Pre-publication history

The pre-publication history for this paper can be accessed here:

http://www.biomedcentral.com/1472-6963/12/297/prepub
